# Should Networks Supplant Tree Building?

**DOI:** 10.3390/microorganisms8081179

**Published:** 2020-08-03

**Authors:** Rob DeSalle, Margaret Riley

**Affiliations:** 1 Sackler Institute for Comparative Genomics, American Museum of Natural History, Central Park West at 79th Street, New York, NY 10024, USA; desalle@amnh.org; 2 Department of Biology, University of Massachusetts Amherst, 116 North Pleasant Street, Amherst, MA 01003, USA

**Keywords:** bacterial phylogeny, horizontal gene transfer, networks

## Abstract

Recent studies suggested that network methods should supplant tree building as the basis of genealogical analysis. This proposition is based upon two arguments. First is the observation that bacterial and archaeal lineages experience processes oppositional to bifurcation and hence the representation of the evolutionary process in a tree like structure is illogical. Second is the argument tree building approaches are circular—you ask for a tree and you get one, which pins a verificationist label on tree building that, if correct, should be the end of phylogenetic analysis as we currently know it. In this review, we examine these questions and suggest that rumors of the death of the bacterial tree of life are exaggerated at best.

## 1. Introduction

There is continuing debate about the impact of horizontal gene transfer (HGT) in our ability to infer phylogenetic relationships among bacteria and archaea. Some recent work on this topic concluded that the death of a bacterial tree of life is a fait accompli [1,2,3,4,5,6,7,8,9] or, less drastically, that a bacterial tree of life is really a “forest of life” [10]. Others argue that tree thinking in bacterial evolutionary biology is like accepting “the tree of 1%” [11,12,13], implying that HGT is so prevalent that it impacts phylogenetic signal in 99% of the genetic elements used to infer phylogeny. 

Specifically, the argument is that some organisms experience processes oppositional to bifurcation and hence the representation of the evolutionary process in a tree-like structure is illogical. To quote one set of authors, the “inevitable noise that creeps into phylogenetic estimations, will all create patterns far more complicated than those portrayed by a simple tree diagram” [1]. The authors who hold this view suggested that network methods should supplant tree building as the basis of genealogical analysis. This conclusion is indeed an evocative and important perspective if warranted. Hence, we examine the premise and logic behind this perspective in this review.

## 2. HGT is the Hobgoblin of Bifurcation or Vertical Divergence

HGT has been referred to as the hobgoblin of bifurcation [14], although we note that hobgoblins are not evil and malicious like goblins, they simply cause mischief and disarray. This vertical lineage disruption is the primary reason some argue that a strictly bifurcating tree of life should be shunned [1,15]. It is correct that HGT disrupts “true” phylogenetic signal and when it occurs one might be able to infer a network of gene relationships, but clearly not a bifurcating tree. This argument has been proposed as a real deal breaker for bacterial and archaeal lineages because these groups are thought to have experienced large amounts of the lineage confusing process of HGT [15,16,17,18,19,20,21].

A slightly different argument has been raised by others who suggested that tree building methods give a tree even when the true evolutionary history is not bifurcating [17]. Clearly, evolutionary processes and optimality criteria must be considered when attempting to reconstruct a phylogeny or, if one prefers, a net in constructing a network. If the processes of bacterial and archaeal divergence violate the basic assumptions of tree building, then we agree that tree building should not be used as an explanatory tool. In this context, we suggest that the following issues need to be examined in a critical and encompassing way. First, we need to detail what we know about the divergence process of bacteria and archaea. Do properties of their overall divergence violate the assumptions of bifurcation? One process, HGT, does occur, and it does, to a certain extent, violate the assumptions of bifurcation. But how often and how much of an impact does HGT have on the divergence process? In addition, in this context, if tree-based analyses truly obscure patterns produced by HGT, then we would also agree that tree approaches should be abandoned. Finally, we need to consider whether using a tree as a null hypothesis is a more or less sound scientific approach, than assuming that a network better explains the data. In this chapter, we examine these questions and suggest that the rumors of the death of the bacterial tree of life are exaggerated at best.

## 3. Bifurcation as an Evolutionary Pattern

The formalization of a preference for bifurcation as a major process in organic evolution comes from Darwin. His “principle of divergence” embodies Darwin’s perception of how species form and diverge during the evolutionary process [22]. Mayr [23] then set the stage for how we might look at evolution in sexually reproducing populations—by coining his biological species definition, which also implies a bifurcating pattern of divergence. The concept of genetic isolation as a means to delineate species arises from this definition and can also easily be applied to clonally reproducing organisms. Simply put, the principle of divergence leads to bifurcation, and Darwin’s preferences for this mode of divergence is embodied in the only figure in *On the Origin*, which is a bifurcating diagram. The simple fact that Darwin preferred this mode of divergence does not mean that we have to accept it though.

The argument made by opponents of a bifurcating bacterial tree of life suggests that the seminal approaches to understanding how life on our planet diverged simply do not hold for bacteria, archaea and even for some deep relationships in the eukaryotic tree of life [5,9,15,16,17]. These opposing views cite the fact that randomly generated data with no background pattern of bifurcation will give a bifurcating tree when methods that force a tree as a solution are used. Hence, the suggestion that tree building is verificationist has arisen.

We point out that there are two approaches that we can take to look at this problem. First, some have suggested that the processes of divergence and speciation in bacteria and archaea are so radically different that using methods to recover bifurcation are illogical. We suggest that a detailed examination of the divergence process in bacteria and archaea can tell us whether or not this conclusion is warranted. By asking, do bacteria and archaea diverge in patterns that negate bifurcation as a valid explanatory process, we can test whether there is sufficient evidence to warrant the exclusion of bifurcation as an explanation. Second, we contend that tree building in the context of phylogenetic analysis does not necessarily result in a tree. In fact, some approaches use a star phylogeny as a null hypothesis and there are some studies where the existence of monophyly (the central idea of tree building) of groups of organisms can be tested [24,25]. In addition, simple tests that allow one to determine if a dataset results in an interpretable bifurcating tree for the tree of life exist [26].

## 4. Bacterial and Archaeal Divergence: Nothing Special?

Data from bacterial genomic studies have led to questions about the appropriate unit of evolution in bacterial biology [27]. Most microbiologists accept that species-level descriptions are both useful and necessary. However, the applicability at the bacterial level of the ‘universal species concept’, with its emphasis on reproductive isolation, has not been universally supported [28,29,30]. At one extreme, some have argued that high levels of HGT make any notion of species simply nonsense. At the other extreme, HGT is dismissed as mere noise. This may be a case where neither extreme captures the interesting aspects of the emerging picture. Instead, we might be best served by asking three interrelated questions: (1) what is the extent and pattern of non-genealogical sharing of genetic information? (2) Are particular classes of genetic information more or less likely to be involved in such exchanges, and (3) is the extent of such non-genealogical sharing sufficient to overwhelm the signature of vertical inheritance?

The ability of bacteria to acquire genetic information in unconventional ways is well-established [11,31]. Further, whole genome sequencing reveals genetic exchange among even distantly related organisms [32,33]. The surprise that the genomic revolution has revealed is thus not the existence of such genetic traffic, but its unexpected range and frequency, which is widespread across bacterial strains. But are these events sufficiently ubiquitous that they require us to abandon the fundamental unit of biological organization, the species?

This question is not merely a semantic one, but rather an empirical challenge. Vertical inheritance is still taking place, leaving behind a genomic signal that we can, in principle, retrieve. Horizontal gene transfer also leaves behind a retrievable signal. The question remains, however, are the phylogenetic reconstructions made possible by rich genomic datasets descending into chaos, suggesting that the noise created by HGT is swamping out the phylogenetic signal? In our view, the signal of vertical inheritance remains loud and clear. In the majority of cases where multiple genes and/or genomes have been used for phylogenetic reconstruction, the resulting trees are resolved and largely congruent [34,35,36]. Furthermore—with some interesting exceptions—these reconstructions match established patterns of classification. 

The availability of whole genome sequences provided our first glimpse into the dynamic nature of a species genome. Glasner and Perna [37] and Mau et al. [38] compared six complete genomes of *Escherichia coli* and revealed a highly conserved genome backbone with greater than 98% sequence similarity among the isolates. However, this conserved backbone was interrupted by hundreds of strain-specific ‘sequence islands’. These patterns of shared and unique sequences appear to be common among bacterial species [39,40,41,42]. However, the relative fraction of the genome shared varies greatly from one bacterial species to the next [43,44,45,46,47]. A highly robust phylogenetic tree was constructed for 13 gamma-proteobacteria using a concatenated alignment of several hundred conserved orthologous proteins [48]. Only two of the proteins had incongruent tree topologies in this analysis. A similar type of investigation was undertaken with Neisseria [49], which revealed that the use of concatenated sequences buffered the distorting effect of recombination events and resulted in the resolution of clusters corresponding to the three most abundant species in the sample. Genome sequence comparisons among members of Agrobacterium highlighted a broad range of intra-species divergence within very closely related but distinct species [50,51,52]. Their data supported earlier claims by Majewski [53] that ‘bacterial species experience a degree of sexual isolation from genetically divergent organisms since recombination occurs more frequently within species than between species’. Konstantintidis and Tiedje [54] compared the gene content of 70 closely related bacterial genomes to identify whether species boundaries exist. They found the levels of sequence similarity on the order of 94% corresponded to the traditional 70% DNA–DNA reassociation standard of the current species definition. As more extensive whole genome data come online, we can expect phylogenetic reconstructions to stabilize. Some existing phylogenetic reconstructions will stand, and others will fall.

Finally, several recent studies have demonstrated that bacterial lineages follow bifurcating or vertical divergence [29,30,55,56,57,58]. Two studies in particular have shown that the dynamics of bifurcation are common and retrievable when considering bacterial lineages. Bobay and Ochman [29] showed that the footprints of divergence are easily detected in all domains of the tree of life. Using measures of gene flow, they showed clearly that bacterial and archaeal lineages are no different from other organisms in showing discontinuities in gene flow. They took this result as evidence that discontinuity in genetic similarity during the divergence process in bacteria conforms to a biological species concept. Jain et al. [30] used the average nucleotide identity (ANI), another method of assessing genomic similarity, to examine species divergence in thousands of genomes (involving billions of comparisons). Intriguingly, they discovered an easily discernable and robust gap between 95% intra-species and 83% interspecies ANI values. This gap is again indicative of divergence in accordance with vertical divergence or splitting. 

Other researchers argued for pluralism in how the divergence process is viewed. Suárez [59] suggested that speciation in the same mode as animals and plants can only be inferred if it can explain divergence better than a null hypothesis based on a random birth death process. They argued that “only when the real data are statistically different from the expectations under the null model that some speciation process should be invoked”. Their assumption is that selection drives speciation and should manifest itself in a statistically significant difference from the null hypothesis. This argument, however, does not recognize random processes such as drift as a means to divergence and hence speciation. 

## 5. Tree Thinking, Concatenation and Bacteria

With the advent of genomic techniques, more and more gene partitions can and have been used in phylogenetic analysis. This has led some researchers to suggest that concatenation of these genes sours the discovery of phylogenetic relationships [15,18]. In essence, some researchers have suggested that gene sequences in a concatenated approach to phylogenetic systematics is a verificationist endeavor [17,18], the sole aim of which is to increase the appearance of support for a phylogenetic branching diagram [60]. We point out here that this view misses the Popperian underpinnings of phylogenetic systematics. As Lienau and DeSalle [60] (p. 195) pointed out:

“The goal of the total evidence approach to phylogenetic research is based in the idea of increasing explanatory power over background knowledge through test and corroboration, rather than to bolster support for nodes in a tree. In this context, the testing of phylogenetic data is a falsificationist endeavor that *includes* [italics added] the possibility of not rejecting the null hypothesis that there is no tree-like structure in molecular phylogenetic data.”

In addition, over the past three decades, phylogenetic studies have recognized the hypothetico-deductive nature of tree building. Maddison [61] recognized that lack of resolution could arise in nearly any phylogenetic analysis. He discussed the lack of resolution as polytomies and coined the terms hard and soft polytomy. A hard polytomy is one that arises as the result of actual trifurcation or even polyfurcation events in the divergence of organisms. A soft polytomy, on the other hand, is one where there simply are not enough data to support one bifurcating pattern over another. If the support for a node is low or even nonexistent, then it can be for two reasons. First, there simply may be no information in the dataset to resolve the node. In this case, there should be no conflicting signal amongst different sources of data. The second reason might be because of direct conflict amongst phylogenetic information, the very reason that we should be wary of HGT. The conflicting signal will manifest itself as a node with low support. In addition, statistical approaches in the phylogenetic comparative method use a star phylogeny as a null hypothesis, explicitly testing for “treeness” [62,63].

**Figure 1:**
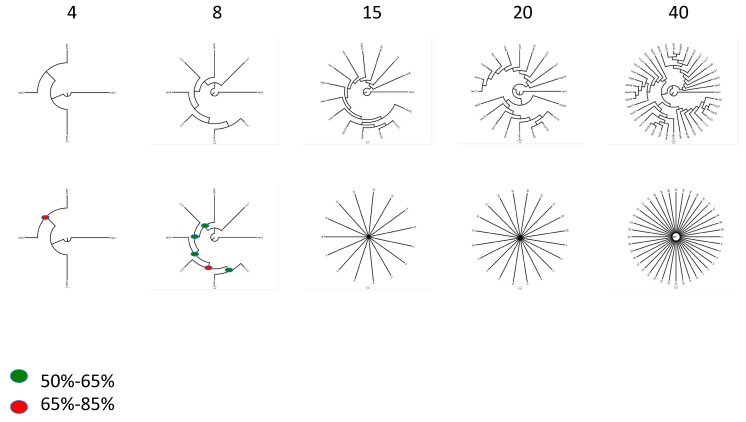
Results of the Ithink experiment. The number of taxa for each part of the experiment is given above the trees. The top row shows the most parsimonious tree obtained for each matrix and the bottom row shows the bootstrap trees for each of the five experiments (NTAX = 4, 8, 15, 20 and 40). Red dots indicate nodes with BP between 66% and 85%. The green dots indicate nodes with BP between 50% and 65%. Construction of the Ithink matrices is described here. We first extracted the text from Darwin’s *On the Origin of Species*. The first 100 letters of the text were turned into a line corresponding to taxon 1, the next 100 letters are turned into a line corresponding to taxon 2, the next 100 letters are turned into a line corresponding to taxon 3 and the next 100 letters are turned into a line corresponding to taxon 4. The process starts over again for the four taxa with the next 100 letters and so on for 100 partitions. The next step is to remove spaces and any letters in the alphabet that do not correspond to an amino acid. These are replaced with ambiguous X’s. Next the individual lines for each partition are transformed into FASTA files and aligned using MAFFT [67] with default settings. Finally, the matrices are formatted for phylogenetic analysis and analyzed using PAUP [68]. We used parsimony as an optimality criterion and weighted all characters equally. The searches were accomplished using 200 rounds of random taxon addition with tree bissection reconnection (TBR) branch swapping. We focused the analyses to be very simple and point out that results using parsimony here will indeed be different from those using likelihood or Bayesian approaches. We obtained bootstrap proportions for the trees shown here using the “boot” option in PAUP with 100 replicates. The “ithink” matrix is available from the authors upon request.

While the suggestion that random data will produce bifurcating trees with relatively good support is true [64,65,66], this phenomenon is very dependent on the number of taxa. As a simple demonstration of this problem, we have created several random phylogenetic datasets by transforming Darwin’s *On the Origin of Species* into amino acid sequences, with assignment of hypothetical taxa to random blocks of text. When matrices with 4, 8, 15, 20, and 40 taxa with 100 genes each (genes with length 100 amino acids) are generated in this way, phylogenetic analysis yields resolved trees. While matrices with 4 and 8 taxa show relatively high support at nodes, the effect erodes as more taxa are allowed to fill the “fake” phylogenetic matrix. By the time 40 taxa are generated, parsimony trees are still resolved but with extremely low or no support measures at the majority of nodes in the tree (Figure 1). We take this as evidence against the claim that random data will produce trees if you ask them to. This brings in to focus the need for assessment of robustness as a part of answering the question does a tree arise from a particular dataset? This leads us to three direct tests for a bacterial tree of life.

## 6. Three Simple Falsificationist Hypotheses that Test for the Existence of the Tree of Life

Lienau et al. [26] proposed three simple falsification-prone hypotheses to test whether a tree of life can be rejected as a means of explaining the divergence of bacterial and archaeal lineages (Table 1). The last hypothesis in Table 1 is the most complex of the three because it requires the testing of several sub-hypotheses based on background knowledge that microbial systematists have established over the last century. These largely revolve around monophyly of well-established groups of organisms and bacterial species and higher category boundaries established by systematists.

It is not surprising to us that all three levels of null hypotheses can be rejected [26,69,70,71,72], indicating that the conclusion that a bacterial tree of life is a good explanation for how life has diverged is valid. This does not mean that HGT and other lineage blurring processes do not occur. On the other hand, this also does not imply that strictly bifurcating tree building methods obscure the role of HGT and lineage sorting (see below).

## 7. Obscured Pattern or Obscured Process

Several authors have claimed that tree-based phylogenetic analyses obscure the discovery of patterns that are of interest to evolutionary biologists. They further suggested that “many tree-based approaches to resolving the evolutionary analysis have been tried, but with little success” [1] (p. 440). They cited two specific examples where they suggest tree-based approaches have “misled” overall interpretation of the evolutionary history of the organisms involved. The first is most relevant to this discussion and comes from the now classic Rokas et al. [73] study on yeast phylogenetics using a genome level dataset. A recent analysis of the yeast dataset disavowing a tree-based approach and preferring a network approach suggested that the disparity in gene tree topology may be the result of genome hybridization [74]. However, standard tree-based approaches [75,76,77] concluded that the incongruence of different gene geneologies as generated from the dataset is easily explained and expanded upon using a bifurcating framework. First, hidden support [76,78,79] can be used as an explanatory tool for the large amount of incongruence. The genes that are involved in incongruence can be identified and a quantitative framework for their behavior can be developed [76]. Second, the impact of outgroup choice is critical in analyzing genome level data. Gatesy et al. [79] showed for the Rokas et al. [73] dataset that inappropriate outgroup choice can result in random rooting and result in seemingly incongruent phylogenies (see also [77]). In fact, Gatesy et al. [79] obtained the same unrooted network for all 106 genes in the dataset indicating that it is only when the unstable root is applied to the networks that incongruence appears. In addition, it depends on the network approach that is applied. Some network approaches are entirely non-phylogenetic [80,81].

## 8. Why a Tree of Life Infected with HGT Still Bifurcates

Gogarten and Townsend [82] pointed out that the impact of HGT on phylogenetic inference is very context-dependent. They suggested that since the incidence of HGT varies among genes and groups of organisms, the impact or effect of HGT will then vary from phylogenetic problem to problem. It is difficult to argue with this important observation, but a detailed examination of the impact of HGT on phylogenetic analysis in some specific examples can show some of the range of the effect HGT might have on phylogenetic analysis.

**Figure 2:**
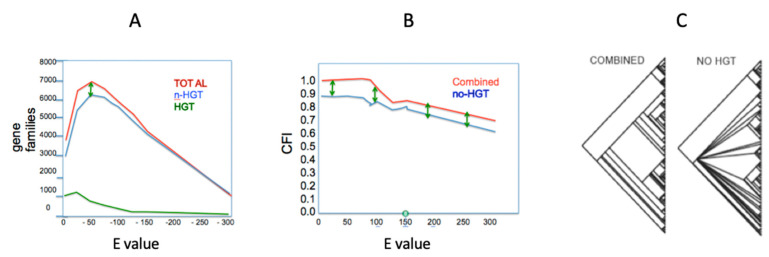
Three results of categorizing gene families as horizontal gene transfer (HGT) or noHGT (see [83] for details). (**A**) The graph represents the plot of E value against number of gene families. Green represents the total number of HGT families, blue represents the total number of noHGT gene families and the red line the total number of gene families at each E value. Most of the impact of HGT occurs at E values less than −100 and the green arrows indicate the difference in nonHGT and total gene families; (**B**) the graph represents a plot of E value versus the consensus fork index (CFI), a measure of consistency of trees in a dataset. To calculate the CFI, the concatenated tree was used as a standard; (**C**) the impact of removing HGT genes from the dataset on resolution of a phylogenetic tree. When HGT genes are removed from the dataset, basal nodes are deresolved. Reference 84is available from the authors on request.

By examining the behavior of data in a phylogenetic context on a node-by-node and character-by-character basis, the impact of HGT on treelike structure can be examined. DeSalle et al. [83] established simple character reconstruction criteria to classify all identifiable orthologs as either impacted by HGT or not (Figure 2). Removal of genes affected by HGT shows three things. First, the proportion of HGT genes in phylogenetic analysis of these 160 genomes relative to non-HGT genes is about 1:7. This ratio was tested at a wide range of E-value cutoffs and it appears to be immune to manipulation of cutoff values. Second, the consistency of a phylogenetic analysis is impacted by the removal of HGT genes. This is a totally unexpected result, as the whole rationale for removing such genes is that they are inconsistent with an analysis. Third, the removal of HGT genes decreases the resolution of a phylogenetic hypothesis at many nodes in an overall tree. This result is obtained because the HGT genes actually carry phylogenetic information relevant to the collapsing nodes. Using similar approaches based on phylogenetic incongruence called Prunier, Abby et al. [71] examined over 350 genomes in 16 bacterial and archaeal phyla for over 12,000 orthologs. They pointed out that most branches in the tree of life they constructed experience an average of 5 to 10% HGT. Zamani-Dahaj [84] used a similar presence absence approach to Desalle et al. [83] for cyanobacteria and archaea and come to the conclusion that for cyanobacteria, about 15% and, for archaea, between 20% and 39% of genes show patterns of HGT.

While there are some groups that experience a large amount of HGT, even these groups are not impacted phylogenetically, forcing them to conclude that “the impact of LGT (HGT) on the branches of the tree of life is significant but not overwhelming” [71,85,86].

**Figure 3:**
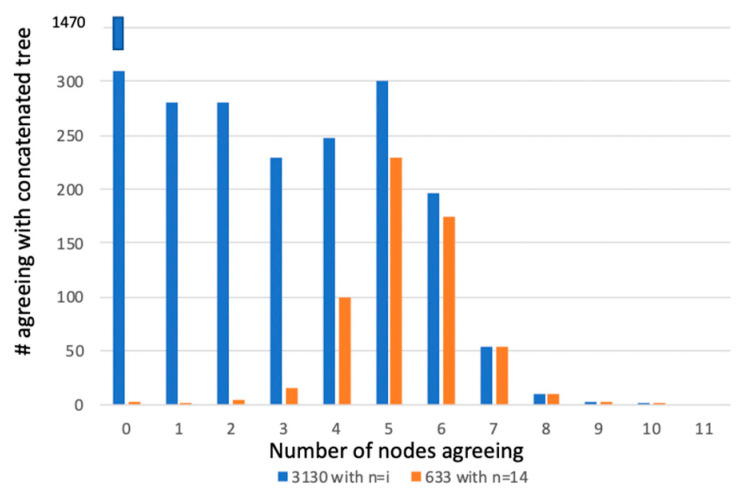
Plot of number of proteins in two different datasets on the Y axis versus the number of nodes in agreement with the concatenated tree. The blue bars represent the dataset with all 3130 proteins, regardless of taxonomic coverage. The orange bars represent the dataset with proteins across all 14 taxa. A 50% bootstrap cutoff was used to say that a node existed for a particular gene partition. See reference [89].

We comment here too about the tree of 1%, first articulated by Dagan and Martin [87]. It is difficult to argue with the premise of the tree of 1%. Of course, if 30 genes are chosen for an analysis and there are 3000 genes in an average bacterium or archaeon genome, then 1% is correct. But some studies used the entire repertoire of proteins from the organisms under study for phylogenetic analysis, and the Lineau et al. [69] dataset shows that we can reject the three hypotheses listed in Table 1. Another example is from di Bonaventura et al. [88], where a phylogeny of 14 species of Pasteurellaceae was placed into a phylogenetic context. Two datasets were considered in this study—first, all 3130 proteins were included regardless of taxonomic overlap, and second a matrix with 633 proteins where all fourteen Pasteruellaceae had the sequence. There are 11 nodes in the concatenated tree for both datasets that are recovered regardless of method and at 100% bootstrap support. Figure 3 shows the number of nodes agreeing with the concatenated trees graphed against the number of proteins in the two datasets for the node number in agreement. The figure demonstrates rampant incongruence in both datasets. Yet a robust and taxonomically reasonable hypothesis is attained that mirrors the current taxonomy of the group. The concatenated phylogeny is the product of the interaction of all of the phylogenetic signal of all of the proteins in the datasets. 

An explanation for this seemingly strange result came from Lienau et al. [26], who pointed out that even if incongruence at a node exists for a particular gene on a global level, there can be hidden support [75] for many of the other nodes in a phylogenetic hypothesis. The easiest way to picture this phenomenon is to think of mitochondrial and bacterial 16S ribosomal DNA. Using this gene to address the topology of the tree of life results in a tree radically incongruent with our background knowledge about the relationships of Archaea, Bacteria and Eukarya. The 16S tree places Eukarya within the bacterial clade and specifically close to the Proteobacteria. However, when one looks at the hidden support 16S rDNA contributes to the accepted tree of life hypothesis, the hidden support is immense. We suggest that it is often overlooked that while HGT impacts phylogeny, it does so only at the point of the HGT. History before and after HGT is oftentimes kept intact. 

## 9. Concluding Remarks

A representation of the history of life serves many purposes. While the first and most technical purpose is to show the pattern of divergence of life on the planet, phylogenetic trees and indeed any method that represents this divergence are important for more practical reasons. Phylogenetic trees are becoming increasingly important in community ecology, biogeography, hybrid zone analysis and even population genetics to name just a few evolutionary subdisciplines that are more and more reliant on phylogenies. It is therefore incumbent upon scientists to use the most accurate approaches to represent the pattern of divergence of life on Earth. 

Challenges to the tree of life have been raised on two major fronts as a result of genome level sequencing. Incomplete lineage sorting ISL [58] and HGT [11,12,13,14,15,16,18,20,21] have both been suggested as “treebusters” at certain levels of divergence amongst organisms. It is true that ISL and HGT disrupt vertical signals and they are both extremely interesting evolutionary processes that deserve attention and focus in both phylogenetic and evolutionary studies. The extent of their impact on vertically evolving and bifurcating lineages has been assumed to be substantial enough that new paradigms to replace the tree of life have been suggested [1]. We suggest that beyond supposedly high levels of HGT, bacteria and archaea play by the same evolutionary rules that involve mutation, recombination, drift and selection. There is nothing really special about their divergence other than HGT, and if HGT either exists at a lower level than is thought or can be shown to be surmountable in the context of phylogenetic analysis, then there is no reason to “uproot” the tree of life. 

Attempts at empirically determining the level of HGT in datasets range between below 15% [71,83,84] and the high set by the tree of 1%. If the tree of life is a “tree of 1%” then the rest—99%—can be used as a limit of non-vertical information [12,13] of the genes in bacterial and archaeal datasets. If we consider those estimates made from presence/absence approaches, the frequency of potential HGT tops out at 35% for archaea and lower for other lineages. From these estimates, we suggest that the level of HGT is not enough to destroy, let alone damage, recovering bifurcating history, especially when we take the lower end of the HGT estimate (5 to 15%) of the genes involved in HGT in a dataset that could actually contribute to the overall phylogenetic signal via hidden support [71,83,84]. We want to make it very clear that we are not minimizing, ignoring or deflecting HGT as a biological process. It exists and it is an important factor in how many microbes have diverged and evolved. We simply suggest that it is not pervasive enough to fell a bifurcating tree of life.

Many readers will be asking why not just use networks to do our analyses in the first place. Such methods can detect tree-like structures very simply and because they are more flexible in their interpretation, they should be preferred over strictly bifurcating trees. We suggest that this approach puts the cart before the horse because using the tree of life as a null hypothesis to examine the impact of HGT and lineage sorting in the tree of life makes fewer and simpler assumptions than assuming a network. In fact, we contend that phenomena such as specific HGT events in an evolutionary context are not discoverable without a tree of life. In essence, we can make the same argument we mentioned earlier, that can be made against tree-based approaches. If you look for a tree with incongruent data, you will find one. We suggest that if you look for a network with incongruent data, you will also find one. Because incongruence can be the result of many things, the relevant question is: do the incongruent data specifically produce a net? We can test hypotheses about the physical mechanisms of HGT, but their evolutionary history can be most efficiently and scientifically tested via tree building methods. This begs the question as to what gene net methods actually tell us that tree building methods do not.

Phylogenetic tree analysis actually allows the researcher to identify HGT events [71,85]. By accepting a concatenated hypothesis for the tree of life, a baseline bifurcating pattern is discovered. This baseline pattern can then be used to interpret any fragment of DNA, gene or cluster of genes as being in line with vertical history or horizontal transfer [71]. In fact, a tree of life offers what we might call more explanatory power than any other method of evolutionary analysis, because a bifurcating tree makes fewer assumptions about the divergence process as first proposed by Darwin in his Principle of Divergence.

We point out that species and speciation in bacteria would not be interpretable in a biological context without a bifurcating tree of life. As we note above, bacteria and archaea play by the same evolutionary rules as eukarya. Mutation, selection and drift result in genotypic and phenotypic clustering that simply would not result with a tree of 1%. Again, we may turn to Darwin’s perspective,

“Why is not all nature in confusion, instead of the species being, as we see them, well defined? [90]?”

Although Darwin was referring to eukaryotes, numerous studies have revealed clusters of bacterial isolates that share complex phenotypes, and these clusters are often designated as species [89,91,92,93,94]. In fact, Cohan used the existence of these clusters as evidence of bacterial species. “Bacterial diversity is organized into discrete phenotypic and genetic clusters… and these clusters are recognized as species.” [95]. Lan and Reeves [96] proposed the core genome hypothesis (CGH), which starts with the biological species concept [23] and acknowledges the potential impact of HGT on bacterial species. The CGH predicts that a subset of bacterial genes, the core, is present in all, or nearly all, individuals within a species. These are the genes that provide the defining characteristics of a species and are assumed to experience primarily purifying selection, to remove deleterious mutations, and to maintain existing functions. As a species evolves, its core genome will evolve as a complex of co-evolved functions. 

The CGH has dramatically influenced how bacteriologists think about the nature of bacterial species. Prior to the CGH, the strongest argument against the recognition of bacterial “species” was the simple observation of HGT between bacterial lineages. The fact that bacterial species gene pools may not be tightly closed was enough reason for many microbiologists to conclude bacterial species could not survive such exchange. This contradicts the fact that bacteria exist in phenotypic clusters, which many microbiologists recognize as species. Even more compelling, it is becoming clear that these well-defined phenotypic clusters correspond to underlying genotype clusters [97,98,99].

Taxonomy of bacteria and archaea would be impossible too. Several authors have argued that the tree of life is essential to advancing microbial taxonomy [100,101,102,103,104,105,106,107]. These authors recognized the importance of a phylogenetic bifurcating tree of life for the advancement of organizing and naming the millions of species of microbes on this planet. This makes good sense, as taxonomy is based on bifurcation of species. Without bifurcation, divergence in a biological context and every scientific endeavor that uses such divergence becomes meaningless.

## Figures and Tables

**Table 1:** Three hypotheses that can be tested to examine the validity of a vertical tree of life.

**Hypothesis 1**	**A Massively Concatenated Matrix of Genome-Based Information Results in a Generally Unresolved Phylogenetic Tree.**
If this hypothesis can be rejected then,	
**Hypothesis 2**	**The Tree Generated from a Massively Concatenated Matrix is not Robust.**
If this hypothesis can be rejected then,	
**Hypothesis 3**	**The Robust Tree Generated from a Massively Concatenated Matrix does not Make Biological Sense (i.e., is in Conflict with Accepted Taxonomic Knowledge).**
If this hypothesis can be rejected then,	No vertical tree of life exists.
